# Characteristics of pediatric e-referrals through SMARC system: a two-year national analysis

**DOI:** 10.3389/fmed.2025.1665256

**Published:** 2025-11-12

**Authors:** Abdullah A. Alharbi, Mohammed A. Muaddi, Meshary S. Binhotan, Ahmad Y. Alqassim, Ali K. Alsultan, Mohammed S. Arafat, Abdulrahman Aldhabib, Yasser A. Alaska, Eid B. Alwahbi, Hussain A. Moafa, Gassem Gohal, Mohammed K. Alabdulaali, Nawfal A. Aljerian

**Affiliations:** 1 Department of Family and Community Medicine, Faculty of Medicine, Jazan University, Jazan, Saudi Arabia; 2 Emergency Medical Services Department, King Saud bin Abdulaziz University for Health Sciences, Riyadh, Saudi Arabia; 3 King Abdullah International Medical Research Centre, Riyadh, Saudi Arabia; 4 Medical Referrals Centre, Ministry of Health, Riyadh, Saudi Arabia; 5 Department of Emergency Medicine, King Saud University, Riyadh, Saudi Arabia; 6 Department of Pediatric, Faculty of Medicine, Jazan University, Jazan, Saudi Arabia; 7 Ministry of Health, Riyadh, Saudi Arabia

**Keywords:** electronic referral, pediatric healthcare, healthcare coordination, Saudi Arabia, healthcare systems, digital health

## Abstract

**Background:**

Electronic referral (e-referral) systems are increasingly vital for coordinating pediatric healthcare services, yet comprehensive analyses of nationwide implementation remain limited. This study examines patterns, outcomes, and system performance of pediatric e-referrals across Saudi Arabia’s healthcare network.

**Methods:**

We conducted a retrospective analysis of all pediatric e-referrals (*n* = 62,206) processed through the Saudi Medical Appointment and Referral Center between January 2023 and December 2024. Data analysis included referral types, subspecialty distribution, regional patterns, acceptance rates, and temporal trends.

**Results:**

Male patients represented 54.19% of e-referrals. We found a predominance of routine outpatient referrals (56.98%), with urgent and lifesaving cases comprising 32.41%. The overall acceptance rate was 91.52%, with 100% acceptance for lifesaving cases. Internal referrals constituted 82.85% of cases. General Pediatric Care (31.61%) accounted for most e-referral followed by Pediatric Cardiology (12.12%), Neurological Diseases (11.79%) and Neonatal Care (11.21%). Temporal analysis revealed an increase in overall referrals from 2023 (48.74%) to 2024 (51.26%).

**Conclusion:**

This first comprehensive analysis of Saudi Arabia’s pediatric e-referral system demonstrates successful implementation of a coordinated care network with high acceptance rates and effective regional self-sufficiency. The findings provide valuable insights into healthcare planning and resource allocation. These findings offer transferable insights for international healthcare systems implementing pediatric e-referral platforms and digital health initiatives.

## Introduction

Children represent a unique population requiring distinct healthcare needs with specialized resources and expertise beyond those provided for adults (1, 2), making it challenging to ensure timely and adequate access to the necessary healthcare services (3). Over the years, pediatric health conditions have significantly changed including increased obesity (4), type 2 diabetes mellitus (5), anxiety (6), and other chronic diseases requiring specialized and advanced care (7). Due to the developmental changes during childhood, a child’s health and well-being can have long-lasting effects that impact their adulthood (6), influencing their medical conditions and adding an economic burden on the healthcare system (8). Thus, investing in pediatric health and well-being is crucial for healthy, long lives of the population. However, access to specialized pediatric care is still limited (9).

The demand for pediatric specialty care is globally high for both medical specialties (e.g., neurology, cardiology, and endocrinology) (8) and surgical ones (e.g., cardiac and general surgery) (2, 10). Securing access to these specialties is crucial, as it is linked with significant improvement in, for example, survival from non-traumatic cardiac arrest (11), pediatric leukemias (12), lung transplantation (13), and better surgical outcomes (14) compared to non-specialized care. However, obtaining this specialized care for pediatrics remain challenging (15) due to several factors such as specialist shortage, geographical barriers, healthcare system infrastructure limitations, and financial constraints (3, 7, 16). Transferring patients to another healthcare facility can therefore be a potential solution to provide the required access to healthcare services, considering the regionalization approach followed for pediatric care in different healthcare systems (17, 18).

To optimize the transfer of patients, Saudi Arabia deployed a nationwide electronic referral system (e-referral) known as the Saudi Medical Appointments and Referrals Center (SMARC) (19). This modern system manages referrals between healthcare facilities in a national level, bridging healthcare facilities across all regions of Saudi Arabia. This system includes all governmental and the majority of private hospitals in one platform, to efficiently streamline the referral process. This system has demonstrated significant advancements in utilizing digital health solutions to coordinate both internal referrals, where the referring and receiving facilities located at the same region, and external referrals, where the referring and receiving facilities located at different regions (20). It has also revealed successful coordination in patient referrals requiring diverse specialties, including those in need of intensive care units and mental health services (21, 22). Furthermore, SMARC provides a specialized pathway for pediatric patients (<14 years), ensuring timely access to necessary healthcare resources. Nonetheless, a comprehensive analysis of this cohort has not yet been conducted.

Building upon this knowledge, this study aims to investigate the patterns of e-referral requests for pediatric patients within the SMARC digital platform. Specifically, this study will examine the referral type, the requested bed, the requested specialty, the referral destination (internal or external), and the acceptance of these referrals. This analysis will provide an epidemiological profile of the pediatric population, considering the changes in pediatric epidemiology over the years (4–7). Given the global expansion of digital health initiatives, understanding pediatric e-referral patterns through systematic analysis can provide valuable insights for healthcare systems worldwide seeking to optimize their pediatric care delivery and resource allocation strategies. Understanding these patterns can offer valuable insights into pediatric healthcare needs, thereby informing the development of evidence-based preventive strategies and optimizing resource allocation, while also demonstrating the significance of utilizing a nationwide e-referral system for the pediatric population.

## Materials and methods

### Study design and setting

We conducted a nationwide retrospective analysis of pediatric electronic referrals processed through Saudi Arabia’s SMARC system from January 2023 to December 2024. The study encompassed pediatric referrals (ages 0 < 14 years) across the Saudi Arabia’s healthcare network, which is administratively organized into five business units (BUs). These BUs coordinate healthcare services across different regions: the Central BU manages Riyadh and Al-Qassim regions; while the Eastern BU (covering the Eastern region), Western BU (overseeing Makkah, Madinah, and Al-Baha), Northern BU (covering Al-Jouf, Northern Borders, Tabuk, and Hail), and Southern BU (managing Asir, Jazan, and Najran). SMARC operates as an integrated digital platform that facilitates and tracks healthcare referrals throughout the Saudi Arabia’s public and private healthcare facilities.

### Data source and processing

We extracted and analyzed 62,206 pediatric referral records from the SMARC system’s central database. Our data validation process included systematic quality checks for completeness, consistency, and accuracy of all variables. This process ensured data integrity while maintaining all relevant information necessary for comprehensive analysis of referral patterns and outcomes.

### Inclusion and exclusion criteria

To optimize the referral acceptance process, each referral received a unique referral code number that was simultaneously distributed to multiple hospitals. When any facility accepted a referral, the system automatically rejected it at all other hospitals to prevent duplicate appointments.

To maintain data integrity, we meticulously tracked each patient’s referral journey and implemented a deduplication protocol: accepted referrals resulted in removal of all duplicates from the dataset, while for consistently rejected referrals, we retained only one rejection entry to avoid statistical bias. Referrals with incomplete documentation (such as missing medical reports) were classified as “incomplete” and excluded from analysis.

Inclusion criteria: Pediatric patients (aged 0–14 years) with complete referral documentation submitted through SMARC between January 2023 and December 2024. Exclusion criteria: (1) duplicate referrals; (2) incomplete documentation; and (3) non-pediatric cases.

Following data cleaning, we assessed the dataset for missing variables, finding that less than 3% of records contained missing data elements. These incomplete records were also excluded, ensuring our final analysis was based solely on complete and valid referral data.

### Pediatric e-referrals pathway through SMARC system

**FIGURE 1:**
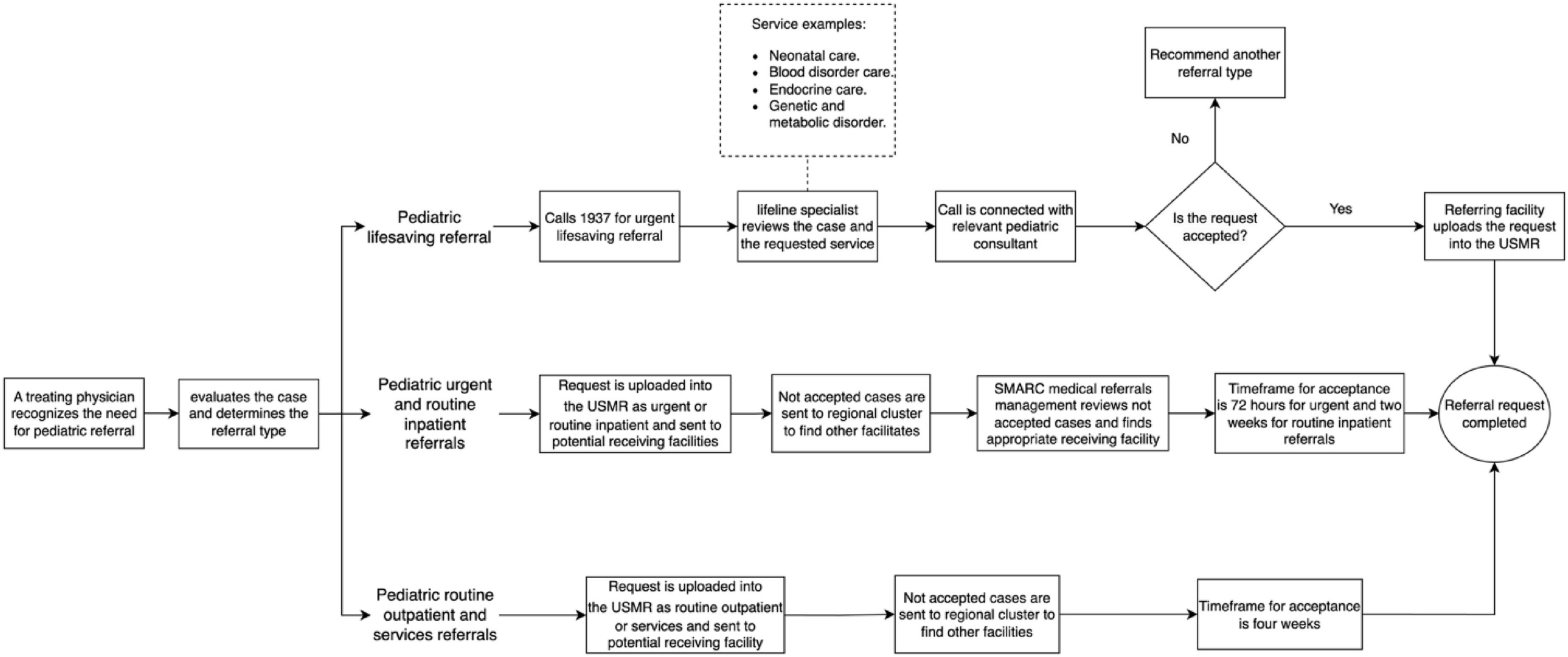
Diagram of pediatric e-referrals pathway through SMARC system (2023–2024).

Figure 1 illustrates the pathway for pediatric e-referrals through SMARC System, starting with the identification of a need for additional resources, proceeding to requesting a referral, and ending by closing the referral request. This Unified System of Medical Referrals (USMR) is digitally accessible by the Management of Appointment Coordination and Medical Referrals (MACMR), which is located in each hospitals, to request a referral.

The referral process initiates when a treating physician recognizes the need for further resources for a child’s medical condition. The treating physician, based on the clinical assessment, determines the appropriate referral type in accordance with the SMARC referral types. The SMARC offers standardized types of referrals based on the patient’s condition, which include urgent, routine inpatient, routine outpatient, services, and lifesaving. Each referral type follows a distinct pathway for requesting, processing, and accepting the request, thereby ensuring prioritized access for patients in most need.

For urgent and routine inpatient requests, both are uploaded into the USMR by the referring facility and sent to the potential receiving facilities where the required resources are available. Not accepted requests are reviewed by the regional cluster to facilitate acceptance. If no acceptance, the case is escalated to the SMARC medical referrals management to further review the request and identify an appropriate receiving facility. For routine outpatient and services requests, they are similarly uploaded into the USMR by the referring facility and sent to the potential receiving facility equipped with the requested resources. Each type of referral adheres to a standardized time for acceptance, as established by the SMARC system: 72 h for urgent referrals, 2 weeks for outline inpatient referrals, and 4 weeks for both routine outpatient and services referrals, starting from the time the request is initiated. Throughout this process, patients continue receiving their medical care at the referring facility until they are transferred to the receiving facility.

For lifesaving referrals, the treating physician contacts the 24-h hotline “1937” for an expedited referral for critically ill patients requiring immediate acceptance. The call is answered by a lifeline specialist who evaluates the request, including the requested service (e.g., neonatal care and pediatric endocrine), and then forward the call to a pediatric consultant in the relevant specialty to comprehensively review the request for immediate acceptance. Accepted requests are provided with the receiving facility’s details and uploaded into the USMR by the referring facility, while rejected ones are advised to be uploaded into the USMR with the recommended referral type.

### Variables and measurements

We examined pediatric referrals across three key domains: patient characteristics, geographical factors, and referral attributes. Patient demographics covered age and sex distributions, while geographical analysis tracked referral patterns across five Business Units (BUs). The referral attributes included multiple dimensions: referral classification (categorized as lifesaving –for critically ill patients requiring immediate referral acceptance according to the treating physician’s assessment of the patient’s medical conditions, urgent–for cases requiring referral within 72 h according to the treating physician’s assessment of the patient’s medical conditions, routine inpatient, routine outpatient, and services–for cases requiring temporary transfer to another facility for specialized investigations such as MRI scans and will be returned to the referring facility); required care level (including outpatient, regular ward, intensive care units - neonatal intensive care unit (NICU) and pediatric intensive care unit (PICU), and specialized units for isolation, cardiac care, and burns); and clinical specialties. The specialty classification included general pediatrics as the core specialty, alongside subspecialties such as cardiology, neurology, gastroenterology, immunology, and hematology. Notably, neonatal care was tracked separately, acknowledging potential overlap with system-specific subspecialties due to its age-based classification. Additional referral parameters included routing patterns (internal within-region versus external cross-regional) and final disposition (acceptance or rejection). Temporal trends were assessed over the 2-years study period.

### Statistical approach

Our analytical strategy combined descriptive statistics with population-based metrics, calculating referral rates per 10,000 pediatric population using current national demographic data. We employed bivariate analysis using chi-square tests to examine relationships between various referral characteristics. Additionally, associations were examined for median age by referral type for pediatric e-referrals through SMARC system (2023–2024) using non-parametric tests including the Kruskal-Wallis test where appropriate, with boxplots for visual clarification. We considered *p* < 0.05 as statistically significant. All analyses were performed using Stata version 16 (StataCorp, 2021).

### Ethical framework

The study protocol received approval from the Ministry of Health’s Institutional Review Board (protocol code 23-77-E and date of approval 20/09/2023). We maintained strict data privacy protocols throughout the study, working with de-identified data sets. Given the retrospective nature of the analysis and the absence of personal identifiers, the IRB waived the requirement for individual consent. Our research methodology adhered to established ethical guidelines for healthcare data analysis.

## Results

**FIGURE 2:**
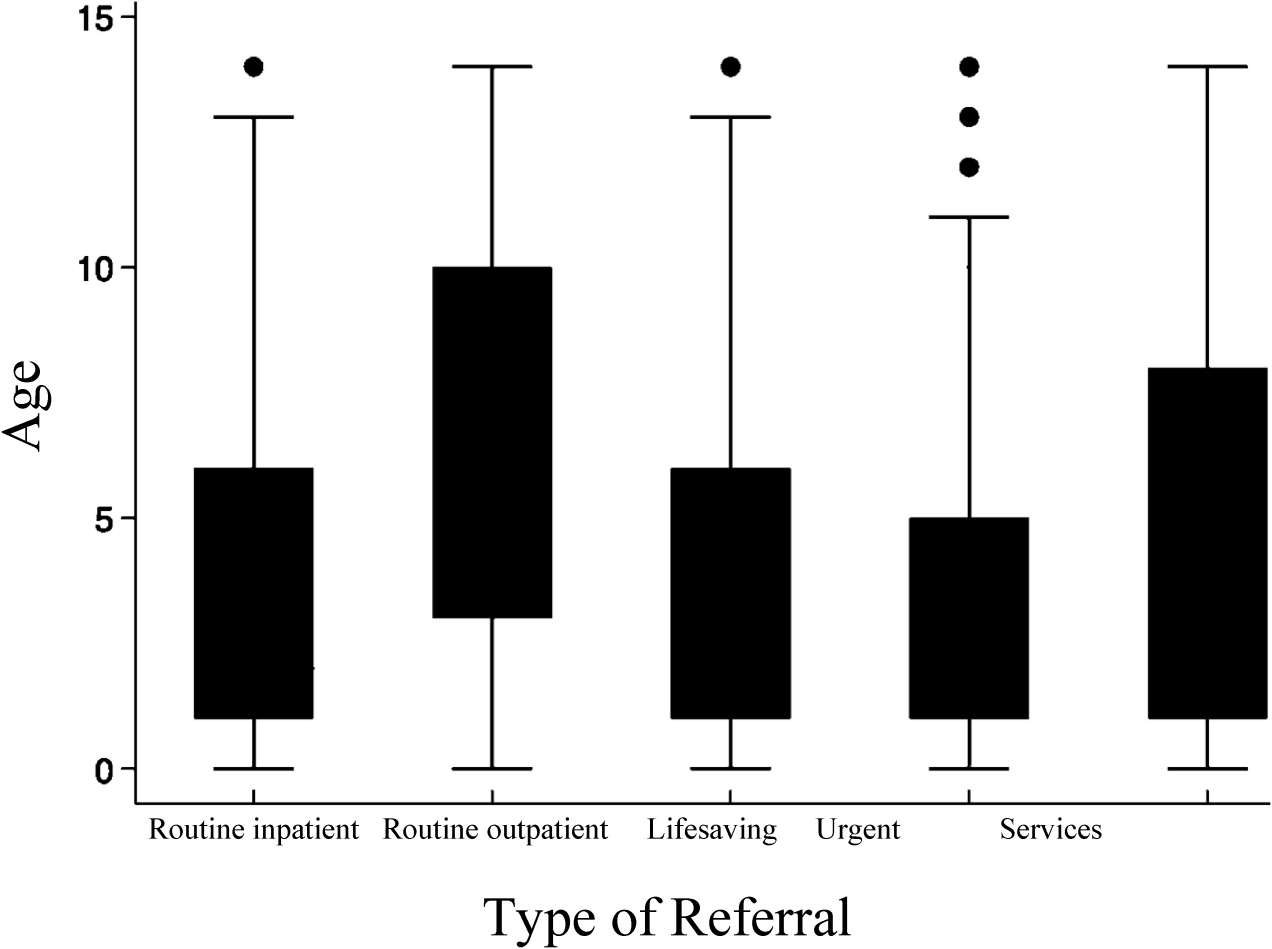
Median age by referral type for pediatric e-referrals through SMARC system (2023–2024).

Analysis of 62,206 pediatric e-referrals revealed distinct patterns in patient characteristics and healthcare needs (Table 1). Demographic analysis showed a male predominance (54.19%) with a mean age of 5.41 ± 4.11 years. The overall referral rate was 77.99 referrals per 10,000 population, with a higher rate among males (83.31 per 10,000) compared to females (72.45 per 10,000). Referral patterns demonstrated that routine outpatient visits comprised the majority (56.98%) of cases, while urgent referrals accounted for 29.60%. Regarding care requirements, most patients needed outpatient services (57.41%), though 21.76% required critical care facilities (PICU/NICU). Age distribution varied by referral type, as illustrated in Figure 2, which depicts median age across referral categories.

**TABLE 1:** Characteristics of pediatric e-referrals through SMARC system (2023–2024).

Characteristics	*N* (%)
Total	62,206 (100.00)
**Age**	
Mean age in years (SD)	5.41 (4.11)
**Gender**	
Female	28,497 (45.81)
Male	33,709 (54.19)
**Year**	
2023	30,320 (48.74)
2024	31,886 (51.26)
**Referral type**	
Routine outpatient	35,447 (56.98)
Urgent	18,411 (29.60)
Routine inpatient	6,189 (9.95)
Lifesaving	1,749 (2.81)
Services	410 (0.66)
**Bed type**	
Outpatient department (no bed)	35,714 (57.41)
Ward bed	12,686 (20.39)
NICU	7,406 (11.91)
PICU	6,129 (9.85)
Isolation bed	254 (0.41)
Burn bed	17 (0.03)
**Referral destination**	
Internal	51,538 (82.85)
External	10,668 (17.15)
**Acceptance status**	
Accept	56,929 (91.52)
Reject	5,277 (8.48)

N, frequency; %, percentage; SD, standard deviation, NICU, neonatal intensive care unit, PICU, pediatric intensive care unit; Data collected from the Saudi Medical Appointments and Referrals Center (SMARC) system during 2023–2024.

Table 2 presents a cross-tabulation of pediatric e-referrals by sex, year, and business unit against five referral types. There was a male predominance (54.19% vs. 45.81%) in pediatric e-referrals and a slight increase in referrals from 2023 to 2024 (48.74% vs. 51.26%). The Western BU handled the highest volume (26.95%) of total referrals, followed by the Southern BU (24.97%).

**TABLE 2:** Demographic and regional distribution of pediatric e-referrals by referral type across Saudi Arabia (2023–2024).

Characteristics	Routine inpatient *N* (%)	Routine outpatient *N* (%)	Lifesaving *N* (%)	Urgent *N* (%)	Services *N* (%)	Total *N* (%)	*P*-value
**Sex**							
Male	3,233 (47.92)	19,900 (56.14)	929 (53.12)	9,430 (51.22)	227 (55.37)	**33,709 (54.19)**	**<0.0001**
Female	2,966 (52.08)	15,547 (43.86)	820 (46.88)	8,981 (48.78)	183 (44.63)	**28,497 (45.81)**	
**Year**							
2023	3,391 (54.79)	16,458 (46.43)	1,095 (62.61)	9,238 (50.18)	138 (33.66)	**30,320 (48.74)**	**<0.0001**
2024	2,798 (45.21)	18,989 (53.57)	654 (37.39)	9,173 (49.82)	272 (66.34)	**31,886 (51.26)**	
**Business unit (BU)**							
Central BU	1,903 (30.75)	7,925 (22.36)	642 (36.71)	2836 (15.40)	1 (0.24)	**13,307 (21.39)**	**<0.0001**
Eastern BU	1,225 (19.79)	3,969 (11.20)	219 (12.52)	1231 (6.69)	268 (65.37)	**6,912 (11.11)**	
Western BU	1,328 (21.46)	8,223 (23.20)	216 (12.35)	6909 (37.53)	89 (21.71)	**16,765 (26.95)**	
Northern BU	524 (8.47)	7,066 (19.93)	208 (11.89)	1888 (10.25)	4 (0.98)	**9,690 (15.58)**	
Southern BU	1,209 (19.53)	8,264 (23.31)	464 (26.53)	5547 (30.13)	48 (11.71)	**15,532 (24.97)**	
**Total**	**6,189 (100.00)**	**35,447 (100.00)**	**1,749 (100.00)**	**18,411 (100.00)**	**410 (100.00)**	**62,206 (100.00)**	

N, number of referrals; Central BU (Riyadh and Al-Qassim), Eastern BU (Eastern region), Western BU (Makkah, Madinah, and Al-Baha), Northern BU (Al-Jouf, Northern Borders, Tabuk, and Hail), and Southern BU (Asir, Jazan, and Najran). Bold values indicate statistically significant p-values (< 0.05).

**FIGURE 3:**
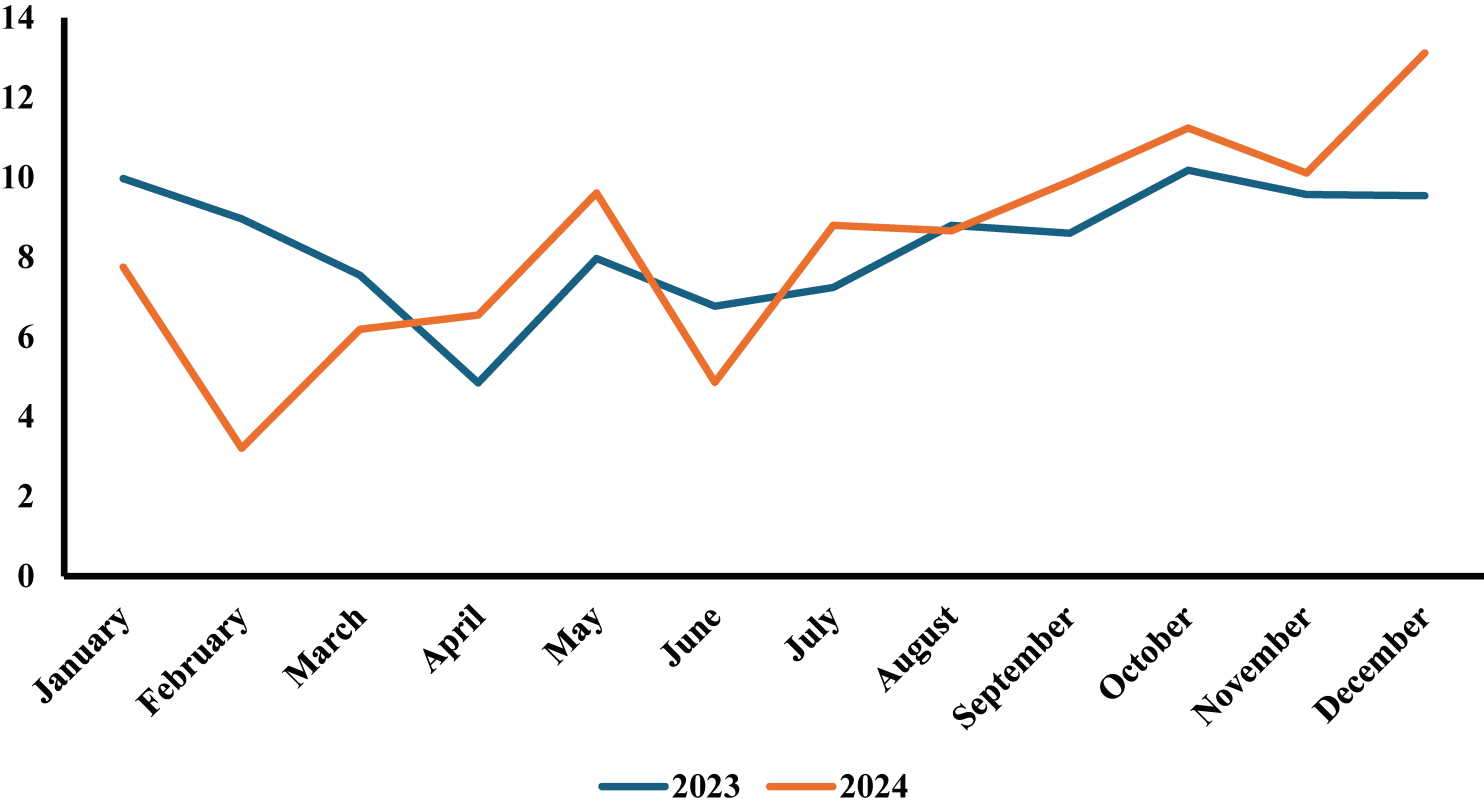
Pattern of percentage monthly pediatric e-referrals requests for the years 2023 and 2024 across Saudi Arabia: comparison between 2023 and 2024.

Figure 3 shows the monthly trends in percentage of pediatric e-referrals across Saudi Arabia during 2023–2024. The two lines illustrate distinct seasonal patterns and year-over-year differences. In 2023, e-referral percentages remained relatively stable, with a noticeable decrease in April 4.85% (1,470 e-referrals) and peak in October 10.18% (3,086 e-referrals). The 2024 pattern showed more pronounced fluctuations, starting with a steep decline from January (7.75%, 2,470 e-referrals) to February (3.21%, 1,024 e-referrals) - a drop of more than 50%. The middle and latter months of 2024 showed generally higher percentages compared to 2023, particularly from September onward. December 2024 reached the highest percentage of e-referrals (13.12%, 4,185 e-referrals) across both years, substantially higher than December 2023 (9.54%, 2,893 e-referrals). The final quarter of 2024 receiving 34.47% of the year’s total e-referrals compared to 29.29% during the same period in 2023.

Table 3 displays the distribution of e-referrals across general pediatric and 14 pediatric subspecialties, broken down by referral type. Pediatric General Care accounted for the largest proportion of referrals (27.34%), followed by Pediatric Cardiology (12.12%), Neurological Diseases (11.79%) and Neonatal Care (11.21%). Urgent referrals were predominantly for General Care (51.89%) and Neonatal Care (26.44%), while Neurological Diseases dominated routine Outpatient referrals (17.62%).

**TABLE 3:** Distribution of pediatric e-referrals by subspecialty and referral type across Saudi Arabia (2023–2024).

Specialty/subspecialty	Routine inpatient *N* (%)	Routine outpatient *N* (%)	Lifesaving *N* (%)	Urgent *N* (%)	Services *N* (%)	Total *N* (%)
Pediatric general care^‡^	2,011 (32.49)	4,656 (13.14)	704 (40.25)	9,554 (51.89)	85 (20.73)	17,010 (27.34)
Neonatal care^†^	1,234 (19.94)	324 (0.91)	521 (29.79)	4,868 (26.44)	27 (6.59)	6,974 (11.21)
Pediatric cardiology	876 (14.15)	5,527 (15.59)	90 (5.15)	970 (5.27)	74 (18.05)	7,537 (12.12)
Pediatric neurological diseases	455 (7.35)	6,245 (17.62)	97 (5.55)	579 (3.14)	68 (16.59)	7,444 (11.97)
Pediatric gastrointestinal and liver diseases	284 (4.59)	2,318 (6.54)	68 (3.89)	417 (2.26)	14 (3.41)	3,101 (4.99)
Pediatric blood diseases	229 (3.70)	1,832 (5.17)	66 (3.77)	449 (2.44)	28 (6.83)	2,604 (4.19)
Pediatric rheumatology	54 (0.87)	294 (0.83)	2 (0.11)	40 (0.22)	6 (1.46)	396 (0.64)
Pediatric endocrine disorders	129 (2.08)	4,147 (11.70)	17 (0.97)	171 (0.93)	12 (2.93)	4,476 (7.20)
Pediatric kidney diseases	199 (3.22)	1,098 (3.10)	43 (2.46)	234 (1.27)	18 (4.39)	1,592 (2.56)
Pediatric respiratory diseases	189 (3.05)	617 (1.74)	82 (4.69)	475 (2.58)	21 (5.12)	1,384 (2.22)
Pediatric infectious diseases	45 (0.73)	82 (0.23)	5 (0.29)	61 (0.33)	28 (6.83)	221 (0.36)
Genetic and metabolic disorders	184 (2.97)	2,967 (8.37)	18 (1.03)	199 (1.08)	21 (5.12)	3,389 (5.45)
Behavioral and growth disorders	7 (0.11)	3,431 (9.68)	0 (0.00)	2 (0.01)	1 (0.24)	3,441 (5.53)
Pediatric oncology	179 (2.89)	304 (0.86)	34 (1.94)	338 (1.84)	1 (0.24)	856 (1.38)
Pediatric allergy and immunology	114 (1.84)	1,605 (4.53)	2 (0.11)	54 (0.29)	6 (1.46)	1,781 (2.86)
**Total**	**6,189 (100.00)**	**35,447 (100.00)**	**1,749 (100.00)**	**18,411 (100.00)**	**410 (100.00)**	**62,206 (100.00)**

N, number of referrals. Pearson Chi-square test showed significant differences in subspecialty distribution across referral types (Chi^2^ = 25330.65, *p* < 0.0001). ^‡^ General pediatrics (core specialty), ^†^ Neonatal care (may overlap with other categories). Bold values indicate total values and statistically significant p-values (< 0.05).

Analysis of referral outcomes revealed significant variations in acceptance rates and destinations across referral types (Table 4). The overall system demonstrated high acceptance (91.52%), with notably perfect acceptance for lifesaving cases and strong performance for urgent referrals (95.49%). Most cases (82.85%) were managed within their originating regions through internal referrals, though urgent cases showed the highest proportion of external referrals (22.56%). Service-related referrals, while comprising the smallest category, maintained high acceptance rate and the highest internal referral destination rates (93.41%).

**TABLE 4:** Acceptance status and referral destination by referral type for pediatric e-referrals across Saudi Arabia (2023–2024).

Characteristic	Routine inpatient *N* (%)	Routine outpatient *N* (%)	Lifesaving *N* (%)	Urgent *N* (%)	Services *N* (%)	Total *N* (%)	*P*-value
**Acceptance status**							
Accept	5,337 (86.23)	31,909 (90.02)	1,749 (100.00)	17,581 (95.49)	353 (86.10)	**56,929 (91.52)**	**<0.0001**
Reject	852 (13.77)	3,538 (9.98)	0 (0.00)	830 (4.51)	57 (13.90)	**5,277 (8.48)**	
**Referral destination**							
Internal	5,271 (85.17)	30,079 (84.86)	1,547 (88.45)	14,258 (77.44)	383 (93.41)	**51,538 (82.85)**	**<0.0001**
External	918 (14.83)	5,368 (15.14)	202 (11.55)	4,153 (22.56)	27 (6.59)	**10,668 (17.15)**	
**Total**	**6,189 (100.00)**	**35,447 (100.00)**	**1,749 (100.00)**	**18,411 (100.00)**	**410 (100.00)**	**62,206 (100.00)**	

N, number of referrals; OPD, outpatient department. *P*-values calculated using Pearson’s Chi-square test, indicating significant differences in both acceptance status and referral destination across referral types (Chi^2^ = 1010.92 and 449.62 respectively, *p* < 0.0001 for both). Bold values indicate total values and statistically significant p-values (< 0.05).

Table 5 showed Pediatric referral data for children aged 0–14 with varying rates across different Business Units (BUs). The Southern BU has the highest referral rate at 135.67 per 10,000 pediatric population, followed closely by the Northern BU at 123.75 per 10,000. The Western BU shows a rate of 67.11, while the Central and Eastern BUs have lower rates at 59.20 and 56.98 per 10,000 respectively. Overall, the national pediatric referral rate stands at 78.87 per 10,000 population, with a total of 62,206 referral requests across all regions.

**TABLE 5:** Acceptance status and referral destination by referral type for pediatric e-referrals across Saudi Arabia (2023–2024).

Business unit	Referral requests *N* (%)	Pediatric population	Rate per 10,000
Central BU	13,307 (21.39)	2,247,790	59.20
Eastern BU	6,912 (11.11)	1,213,128	56.98
Western BU	16,765 (26.95)	2,498,152	67.11
Northern BU	9,690 (15.58)	783,017	123.75
Southern BU	15,532 (24.97)	1,144,803	135.67
**Total**	**62,206 (100.00)**	**7,886,890**	**78.87**

BU, business unit; Central BU (Riyadh and Al-Qassim), Eastern BU (Eastern region), Western BU (Makkah, Madinah, and Al-Baha), Northern BU (Al-Jouf, Northern Borders, Tabuk, and Hail), and Southern BU (Asir, Jazan, and Najran). Population data based on pediatric population aged 0–14 years. Bold values indicate total values.

## Discussion

This nationwide study examined the patterns, distribution, and outcomes of pediatric e-referrals across Saudi Arabia’s healthcare system during 2023–2024, representing the first comprehensive analysis of its kind in the region. Our analysis of 62,206 pediatric e-referrals revealed several key findings: First, routine outpatient referrals constituted the majority (56.98%) of all pediatric e-referrals, while urgent and lifesaving referrals together comprised approximately 29.60%, reflecting the system’s dual role in managing both routine and urgent pediatric care needs. Second, the study demonstrated a remarkably high acceptance rate of 91.52% across all referral types, with notably perfect acceptance (100%) for lifesaving cases, indicating an efficient triage and response system. Third, the distribution analysis revealed that Pediatric General Care constituted the largest proportion of e-referrals (27.34%), followed by Pediatric Cardiology (12.12%), Neurological Diseases (11.79%) and Neonatal Care (11.21%) subspecialties, suggesting opportunities for optimizing care pathways and resource allocation. These findings provide crucial insights for healthcare policymakers and administrators in understanding referral patterns and system performance, while establishing a baseline for monitoring and improving pediatric healthcare services across Saudi Arabia. The comprehensive methodology and analytical framework developed in this study offer a replicable approach for evaluating pediatric e-referral systems in diverse healthcare contexts globally, while the observed subspecialty distribution patterns and system performance metrics serve as comparative standards that can be adapted by other countries advancing their digital health infrastructure.

Our findings regarding urgent and lifesaving referrals (29.60%) contribute to the growing literature on pediatric care coordination through digital platforms. While previous studies have primarily focused on specific aspects such as mental health referrals (23) or emergency department utilization (24, 25), our study provides a comprehensive view of a national e-referral system’s capacity to handle varying acuity levels. The high acceptance rates, particularly for critical cases, align with evidence suggesting that structured digital referral systems can improve care coordination efficiency (26, 27). This is particularly relevant given that previous studies of e-referral solutions have identified clear protocols and robust triage mechanisms as key success factors (28–30).

The subspecialty distribution pattern provides novel insights into pediatric healthcare needs on a national scale. While existing literature has examined subspecialty workforce distribution and access patterns in various healthcare settings (31–36), our findings specifically demonstrate how an e-referral system facilitate subspecialty care coordination. The predominance of General Pediatric Care referrals suggests opportunities to optimize existing telehealth services (i.e., Virtual Clinics Service) and enhance their utilization through improved family awareness and engagement, addressing both system capacity and healthcare-seeking preferences documented in pediatric service research (37–39). The predominant subspecialty distribution aligns with documented increasing complexity in pediatric care delivery (40–42). The 82.85% internal referrals versus 17.15% external referrals demonstrate that most of pediatric specialty care is managed within BUs in Saudi Arabia. However, these e-referrals represent only 0.018% of the nation’s total healthcare activity, which processed over 170 million visits in 2024 alone (43). The selective external referrals reflect strategic concentration of specialized services at centers of excellence, an evidence-based approach demonstrating improved clinical outcomes in pediatric high-volume centers (44). This referral mechanism, supported by MOH infrastructure facilitating access to specialized centers, validates the current strategic distribution model. While our descriptive analysis reveals referral patterns, developing specific optimization strategies requires additional operational data beyond our study scope. Future research should connect patient-level data with facility-level information (such as staffing and resource availability) to better understand regional distribution patterns and inform resource allocation policies, allowing for more comprehensive analyses that incorporate both demand (patient referrals) and supply (healthcare resources) factors across regions. The substantial proportion of neurological, cardiology and neonatal referrals aligns with studies documenting increasing complexity in pediatric care delivery (40–42) and highlights the importance of specialized service distribution in healthcare planning. The variation in subspecialty referral volumes reflects actual disease prevalence rather than sampling limitations. This pattern aligns with epidemiological data, as certain pediatric conditions (like rheumatological disorders) have lower prevalence compared to others, similar to patterns observed in other healthcare systems such as the US. For example, pediatric rheumatology referrals in our study represented only 0.64% (*n* = 396) of total referrals, which aligns with the relatively low prevalence of rheumatological conditions in the pediatric population. According to CDC data, Rheumatoid arthritis affects approximately 305 per 100,000 children in the US (0.305%), illustrating that our referral patterns are consistent with expected disease prevalence (45). Our large national dataset (*n* = 62,206) provides substantial statistical power for this descriptive analysis.

The system performance metrics extend current knowledge about healthcare coordination effectiveness in large-scale e-referral implementations. The high proportion of internal referrals (82.85%) supports findings from previous studies on healthcare decentralization success factors (46), while adding new insights about regional self-sufficiency in pediatric care delivery. The differential rejection rates between routine and urgent referrals (∼10%–14% vs. 3.5%) parallel patterns observed in smaller-scale e-referral studies (22, 23, 47), though our findings demonstrate better urgent case outcomes. This achievement is particularly noteworthy given the challenges of maintaining high acceptance rates in comprehensive e-referral systems. The successful coordination across subspecialties through our e-referral system extends current knowledge about digital platforms’ role in healthcare delivery (23, 26), particularly in supporting decentralized care models while maintaining quality standards for both routine and urgent cases.

International experiences with e-referral implementation highlight the challenges many healthcare systems face. Iran’s e-referral pilot program encountered obstacles including delayed physician payments, inadequate public awareness, and resistance from both patients and providers (48), while the UK’s NHS e-Referral Service achieved 62% electronic processing rates (49). The United States’ Massachusetts e-referral system encountered data standardization challenges, such as difficulty in systematically tracking referral completion (50). China’s Luohu district referral network revealed structural challenges including poor communication between secondary hospitals and other institutions, and significant variations between public and private healthcare facilities (51). In contrast, the SMARC system’s high acceptance rate, with immediate acceptance for lifesaving cases, demonstrates a promising performance in overcoming common implementation barriers that have challenged other international systems, establishing empirical benchmarks that can guide pediatric e-referral optimization efforts worldwide.

The findings of this study have several important implications for public health planning and healthcare policy in Saudi Arabia. The high volume of pediatric e-referrals (62,206) and the substantial proportion of urgent and lifesaving cases (32.41%) underscore the critical role of the e-referral system in coordinating pediatric care. The predominance of internal referrals (82.85%) across all regions demonstrates robust regional healthcare capacity and successful decentralization of pediatric services. The selective use of external referrals (17.15%), particularly for urgent cases (22.56%), likely reflects an appropriate escalation pattern for complex cases requiring specialized care at tertiary centers. The dominance of General Pediatric Care (27.34%) and high urgent referral rates suggest opportunities to optimize existing primary care capacity and implementing clear referral guidelines to optimize resource utilization. The exemplary acceptance rates for critical cases (100% for lifesaving and 95.49% for urgent referrals) demonstrate the system’s effectiveness in prioritizing urgent care. These findings can guide policymakers in maintaining and enhancing the current distributed care model while strategically developing specialized services at tertiary centers for complex cases that genuinely require external referrals.

### Strengths and limitations

This study has several strengths and limitations that should be considered when interpreting its findings. The primary strengths include its comprehensive national scope, covering all pediatric e-referrals across Saudi Arabia’s healthcare system, large sample size, and the inclusion of detailed referral characteristics including types, subspecialties, and outcomes. The 2-year study period allowed for the observation of temporal trends and seasonal variations, while the standardized electronic referral system ensured consistent data capture and minimized missing information. However, some limitations warrant mention. First, the study’s reliance on administrative data means we could not assess the appropriateness of referrals or the clinical outcomes of referred patients. Second, while the study documented acceptance rates and referral destinations, it could not evaluate waiting times, actual service delivery times, or patient/provider satisfaction with the referral process. Finally, the study period coincided with the post-COVID-19 recovery phase, which might have influenced referral patterns and healthcare-seeking behaviors, potentially limiting the generalizability of findings to normal circumstances. Future research incorporating clinical outcomes, patient experiences, and longer follow-up periods would provide additional valuable insights into care pathway completion and patient outcomes.

## Conclusion

This nationwide analysis of pediatric electronic referrals demonstrates the successful implementation and effectiveness of Saudi Arabia’s centralized e-referral system in coordinating pediatric healthcare services. The high acceptance rates, particularly for critical cases, coupled with the predominance of internal referrals, indicate robust regional healthcare capacity and appropriate escalation patterns for specialized care. The clear patterns in subspecialty distribution and referral types, with predominant needs in general pediatrics, cardiology, neurology, and neonatal care, provides an evidence base for strategic healthcare planning and resource allocation. These findings suggest that Saudi Arabia’s electronic referral system has established a robust framework for pediatric care coordination, while also identifying specific areas where targeted service enhancement could further optimize pediatric healthcare delivery across the Saudi Arabia.

## Data Availability

The data analyzed in this study is subject to the following licenses/restrictions: The dataset contains de-identified patient health information from the Saudi Ministry of Health SMARC system and is subject to institutional data governance policies, patient privacy regulations, and ethical approval conditions. Access is restricted to authorized researchers with approved research protocols and signed data use agreements. Data cannot be shared publicly or redistributed without explicit institutional permission and additional ethical review. Requests to access these datasets should be directed to Abdullah A. Alharbi, aaalharbi@jazanu.edu.sa.
